# Femtosecond Laser Fabrication of Submillimeter Microlens Arrays with Tunable Numerical Apertures

**DOI:** 10.3390/mi13081297

**Published:** 2022-08-12

**Authors:** Tongzhen Yang, Minjing Li, Qing Yang, Yu Lu, Yang Cheng, Chengjun Zhang, Bing Du, Xun Hou, Feng Chen

**Affiliations:** 1 School of Mechanical Engineering, Xi’an Jiaotong University, Xi’an 710049, China; 2 State Key Laboratory for Manufacturing System Engineering and Shaanxi Key Laboratory of Photonics Technology for Information, School of Electronic Science and Engineering, Xi’an Jiaotong University, Xi’an 710049, China

**Keywords:** microlens, femtosecond laser-based linear scanning, numerical aperture, optical performance, submillimeter-scale

## Abstract

In recent years, the demand for optical components such as microlenses has been increasing, and various methods have been developed. However, fabrication of submillimeter microlenses with tunable numerical aperture (NA) on hard and brittle materials remains a great challenge using the current methods. In this work, we fabricated a variable NA microlens array with submillimeter size on a silica substrate, using a femtosecond laser-based linear scanning-assisted wet etching method. At the same time, the influence of various processing parameters on the microlens morphology and NA was studied. The NA of the microlenses could be flexibly adjusted in the range of 0.2 to 0.45 by changing the scanning distance of the laser and assisted wet etching. In addition, the imaging and focusing performance tests demonstrated the good optical performance and controllability of the fabricated microlenses. Finally, the optical performance simulation of the prepared microlens array was carried out. The result was consistent with the actual situation, indicating the potential of the submillimeter-scale microlens array prepared by this method for applications in imaging and detection.

## 1. Introduction

With the advancement of micro-nano manufacturing technology, the requirements for microelectronics and optical devices are becoming higher and higher. As miniature optical elements, microlenses have the advantages of miniaturization, light weight, good optical performance, and easy integration. Therefore, microlenses have a wide range of applications such as wavefront detection [1], ultrafast imaging [2], 3D imaging [3,4], virtual reality [5], bionic artificial compound eye structure [6,7], solar microconcentrators, and so on. NA is a very important parameter of the microlens, which represents the imaging and focusing ability of the microlens. When the microlens is applied to different situations, the required NA may be different; therefore, it is important to integrate multiple microlenses, with different NAs, on one sample [8].

At present, various methods for fabricating microlenses have been reported, such as photolithography [9], micro-jet printing [10], nanoimprinting [11], self-assembly [12], and so on. However, the above methods are difficult to control and it is difficult to adjust the NA of the microlens; in addition, most of them are only applicable to polymer materials, while the small size of the microlenses prepared by these methods also limits their application range. Therefore, it is still a challenge to fabricate large-scale microlens arrays with tunable NA on hard and brittle materials.

Femtosecond lasers, as a flexible processing method, have been proven to be able to process any material [13], and are of particular advantage when processing hard and brittle materials [14]. In addition, this method has an ultra-high processing resolution, which can build fine structures on the surface or inside the material [15,16,17]. The femtosecond laser-assisted wet etching (FLWE) method has been demonstrated to greatly improve fabrication efficiency and surface quality, which provides an excellent solution for preparing microlenses on hard and brittle materials [18,19]. In this paper, a femtosecond laser-based linear scanning-assisted wet etching method was used to fabricate microlenses with dimensions close to submillimeter level and flexible NA. Generally speaking, the size of the microlens is closely related to the area of the laser modified zone. Therefore, the influence of laser energy, laser scanning length, and laser scanning speed on the microlens morphology was studied. By changing the power and scanning length of the laser, the area of the modified region was changed, thereby changing the morphology of the microlens to adjust the NA. After testing and calculation, it was found that the NA can vary continuously from 0.2 to 0.45. Finally, multi-NA lens arrays with different arrangements were integrated on the sample. Morphology and optical performance test results demonstrated the flexibility and accuracy of this method.

## 2. Materials and Methods

### 2.1. Femtosecond Laser Ablation

**Figure 1:**
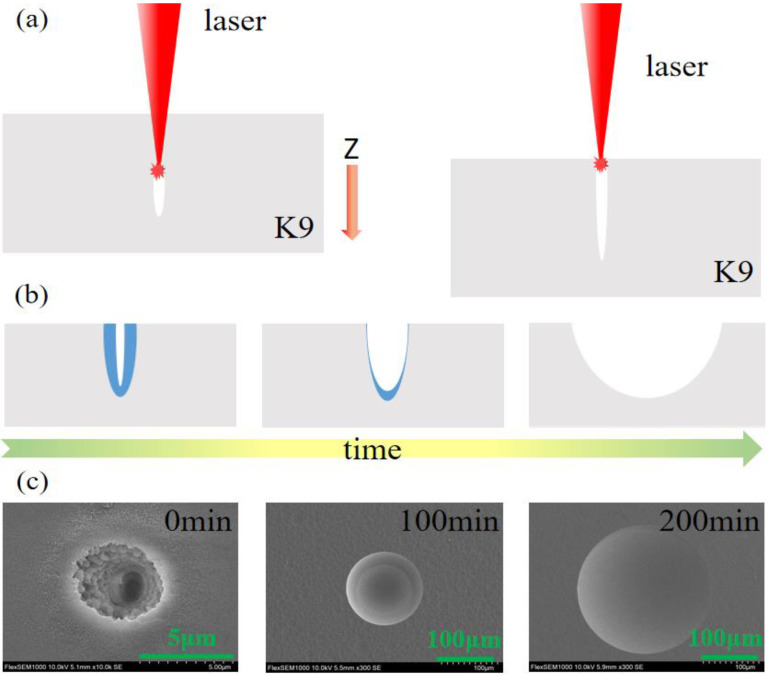
(**a**) Schematic diagram of microlens processing. (**b**) Schematic illustration of the etching process. (**c**) SEM image of microlens as the etching time increases.

The fabrication process of the submillimeter microlens with variable NA is shown in Figure 1. The sample (K9 glass) was fixed on a three-dimensional (3D) translation stage. The femtosecond laser (wavelength = 800 nm, repetition frequency = 1 kHz, pulse width = 35 fs), generated by a Ti:sapphire laser system, passed through a mirror, mechanical shutter, and aperture in sequence, and was then focused on the sample surface using an objective lens (50×, NA = 0.8, Nikon, Tokyo, Japan). As shown in Figure 1a, we used the 3D translation stage to control the relative position of the sample and the laser. First, the laser was focused at a certain depth inside the material. Then, the translation stage was moved downward at a constant speed to change the relative position of the sample and the laser, so that the laser moved vertically upward relative to the sample from the focus point, until it was separated from the surface of the sample. Finally, a narrow microchannel structure was formed inside the sample. We refer to the distance from the initial focus position of the laser to the sample surface as the laser scan length (SL), and the moving speed of the 3D translation stage as the scanning speed (SS) of the laser. During this process, the laser scanning speed and length of the scanning area were controlled by the program.

### 2.2. HF Wet Etching

Different concentrations of hydrofluoric acid (HF) have different selection ratios for the modified area and the non-modified area [20,21]. Based on the requirements for the morphology and imaging capabilities of the lens, 10% HF (diluted from the original reagent solution (aladdin, 40%, GR) with deionized water) was selected as the etching solution for wet etching. The entire etching process was assisted by an ultrasonic bath (CJ-G040SST, SCJ Inc., Harrington, DE, USA) with a frequency of 120 kHz and power of 300 W at room temperature. After etching for about 200 min to 300 min, submillimeter microlenses with good morphology and optical performance can be obtained.

### 2.3. Morphology Characterization

The macro-morphology of the microlens array was captured with a large field microscope (Nikon, SMZ745T, Tokyo, Japan). The detailed surface and cross-section morphology characterizations and analysis were carried out with a scanning electron microscope (Hitachi, FlexSEM 1000, Tokyo, Japan) and laser scanning confocal microscopy (Olympus, LEXT OLS4000, Tokyo, Japan).

## 3. Results and Discussion

### 3.1. Formation of Submillimeter Microlenses

After femtosecond laser irradiation, the inside of the material was laser ablated, resulting in a deep hole and surrounding modified area on the sample surface. Then, wet etching was performed to obtain microlenses. As shown in Figure 1b, the etching process can be roughly divided into two steps [22]. The first step is the corrosion of the modified area. Through the nonlinear effect of the femtosecond laser, the laser injects photon energy into the material, which produces a coulomb explosion [23] inside and triggers avalanche ionization [24,25,26]. This forms a nano-striped structure [27,28,29] on the surface of the material, and produces Lewis bases with greatly enhanced chemical reactivity with acids. Therefore, the corrosion rate of the acid solution to the modified area is much higher than that of other areas. After this process of etching, the modified area is basically removed. The second step is the isotropic corrosion of the overall structure. In this process, the acid solution corrodes in all directions at the same rate. However, because the modified area has a certain depth, as the acid permeates downwards it reacts with the material, causing the acid concentration to drop, so that the corrosion rate at different depths is different. That is, the corrosion rate is inversely proportional to the depth. Therefore, after a certain period of corrosion, the diameter of the lens structure was continuously increasing, but the depth was slowly decreasing and tending to a constant value (Figure S1). In addition, we used scanning electron microscopy to characterize the etching process. Figure 1c shows the etching results of the microlenses at 0, 100, and 200 min. It can be seen that the whole process is consistent with the assumptions, and that the surface of the sample becomes smooth after 200 min of etching. After testing by laser scanning confocal microscopy, its surface roughness was about 21 nm. It is therefore proven that the microlenses prepared by this method have good morphology and quite a high surface quality.

### 3.2. Morphology Control of Microlenses

**Figure 2:**
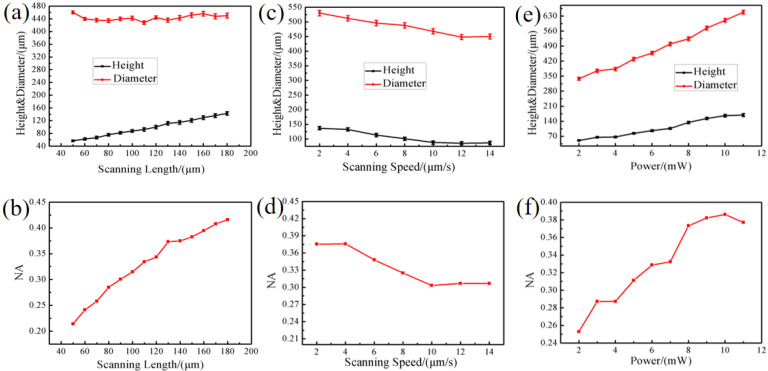
Variation curve of diameter and height of microlens with scanning length (**a**), scanning speed (**c**), and power (**e**) and the curve of the NA of the microlens as a function of scanning length (**b**), scanning speed (**d**), and power (**f**).

The effects of laser SL, SS, and laser power on the morphology and NA value of submillimeter microlenses were explored. For microlenses, NA is a very important parameter which describes the size of the light-receiving cone angle of the microlens, and thus determines the light-receiving ability and spatial resolution of the microlens. After deduction, the calculation formula of NA is: NA = 2 h(n − 1)/(h^2^ + r^2^), where h is the crown height of the microlens, r is the radius of the microlens, and n is the refractive index of the silica (n = 1.46) [30]. Figure 2a shows the variation law of lens height and diameter obtained by changing the laser SL when the power is set as 5 mW and the SS is 10 μm/s. As shown in Figure 2a, the lens diameter at different scanning lengths maintains a certain value, but the lens height increases in a proportional relationship to the scanning length. When the SL is changed from 50 μm to 180 μm, the height of the microlens is increased from 56 μm to 143 μm. Since the SL is changed without changing the laser power during this process, the length of the resulting ablated region changes while the width remains constant. Therefore, the diameter of the microlenses remains unchanged, while the height gradually increases. As shown in Figure 2b, the change curve of NA value also proves that changing the laser scanning distance has an obvious effect on regulating NA value. After calculation, when the SL changes from 50 μm to 180 μm, the NA of the microlens changes from 0.21 to 0.42. 

To explore the relationship between the microlens morphology and the scanning speed, the laser power and the scanning length were set to constant value of 5 mW and 100 μm, respectively. As shown in Figure 2c, both the diameter and height of the microlenses are inversely proportional to the SS. When the SS is changed from 2 μm/s to 14 μm/s, the diameter of the microlens is decreased from 530 μm to 450 μm, and the height from 137 μm to 87 μm. This phenomenon is easy to understand. The repetition frequency of the laser pulse is fixed. Under a certain SL, the lower the SS, the longer the laser action time, the more pulses acting on the material. Therefore, the nanostripe structure of the modified region is more uniform and there are more Lewis bases. This allows for a faster etch rate, resulting in larger microlens structures. Based on the change curve of NA value, it can also be seen that the influence of scanning speed on NA value is not very obvious. When the SS is enhanced from 2 μm/s to 14 μm/s, the NA of the microlens only changed by 0.7 (Figure 2d). However, when the scanning speed is too low, the processing efficiency is too low; and if the scanning speed is too fast, the yield is too low. Therefore, it is necessary to select an appropriate scanning speed. Finally, after comprehensively considering the fabrication efficiency and yield, 10 μm/s was subsequently selected as the scanning speed in the following experiment. Figure 2e shows the variation of the height and diameter of the lens with the laser power under the SL of 100 μm and SS of 10 μm/s. It can be seen that the diameter and height of the lens is proportional to the power of the laser; when the laser power was changed from 2 mW to 11 mW, the diameter of the microlens changed from 338 μm to 648 μm, and the height of the microlens increased from 50 μm to 169 μm, respectively. The change in diameter is easy to explain; the high laser power increases the irradiation range, thereby increasing the area of the modified region. However, under different laser powers, the height of each lens is different. We tested the irradiated surface using a scanning electron microscope (Figure S2). SEM images of the irradiated surface show that the diameter of the ablated region expands with increasing laser power. Therefore, in the subsequent wet etch step, the rate of acid entry into this area increases, resulting in a faster etch rate. While the corrosion rate of each sample surface is the same, the faster the corrosion rate at the bottom, the higher its height. However, the power cannot be increased indiscriminately, because too much power will cause the thermal effect of the laser to be too strong inside the material, causing damage to the material, and affecting the shape of the lens. Similarly, the influence curve of power on NA also shows that the NA of the microlens cannot be adjusted by increasing the power without limit, and the laser power should be selected reasonably on the basis of ensuring the quality of the microlens (Figure 2f).

### 3.3. The Imaging Characterization of MLA with Multiple NAs

**Figure 3:**
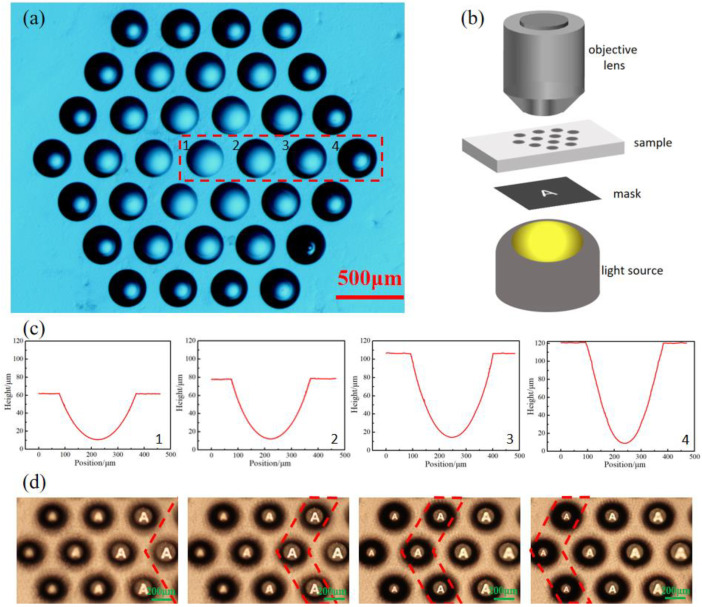
(**a**) Top view of multi-NA submillimeter microlens array under wide field microscope. (**b**) Schematic diagram of the microlens imaging performance testing device. (**c**) Cross-sectional images of microlenses with different NAs, the images from left to right correspond to the microlenses with NA from small to large, respectively. (**d**) Imaging effects of microlens array on different image planes.

After studying the influence of processing parameters on the microlens morphology and NA, microlens arrays with multiple different NAs were prepared on one sample. The laser power was 5 mW, the scanning speed was 10 μm/s, and the laser scanning lengths were 50 μm, 80 μm, 120 μm, and 170 μm, respectively. Figure 3a shows the top view of the as-prepared microlens array that was obtained under a wide field microscope. In order to locate the position of each microlens and determine the processing parameters, the microlenses were arranged in a hexagonal shape. In addition, the microlenses were divided into four groups from the inside to the outside, corresponding to each ring. The microlenses of each ring have the same processing parameters. The SL of the most central microlens is 50 μm, while those located further outwards are 80 μm, 120 μm, and 170 μm, respectively. As shown in Figure 3c, the cross-sectional views of the microlenses numbered 1, 2, 3, and 4 correspond to the numbered microlenses in the top view. In the cross-sectional views of the microlenses, the microlenses with different NAs have the same aperture but different depths. NA is proportional to the height of the microlens. The top and cross-sectional views demonstrate the uniformity and good surface quality of the fabricated submillimeter microlenses.

The imaging measurements schematic is illustrated in Figure 3b, which consisted of a white light source, image target with the letter “A”, the as-fabricated MLA, an objective lens (5×, NA = 0.15), and a Charge Coupled Device (CCD). The MLA was horizontally fixed on a movable platform and could be moved up and down in the direction parallel to the light incidence (“*Z*” direction in this paper). The target was placed between the white light source and the device. Finally, the obtained images were magnified by the microscope objective and then received by using the CCD. By moving the position of the MLA in the *Z* direction to change the distance between the element and the objective lens, the MLA exhibits multi-plane imaging characteristics, as shown in Figure 3d. The z value of 0 μm was recorded when the microlens with the smallest NA in the MLA was most sharply imaged. Continuously moving the MLA to position the remaining microlenses where the sharpest image could be seen, the remaining microlenses had the z-values of 40.3 μm, 100.2 μm, and 151.1 μm, respectively. The test results show that the microlens array can image objects within a vertical range of at least 150 μm. Compared to a microlens array with single NA, the imaging range and fault tolerance are greatly improved, indicating a potential application value in 3D detection.

### 3.4. Focusing Ability of the MLA

**Figure 4:**
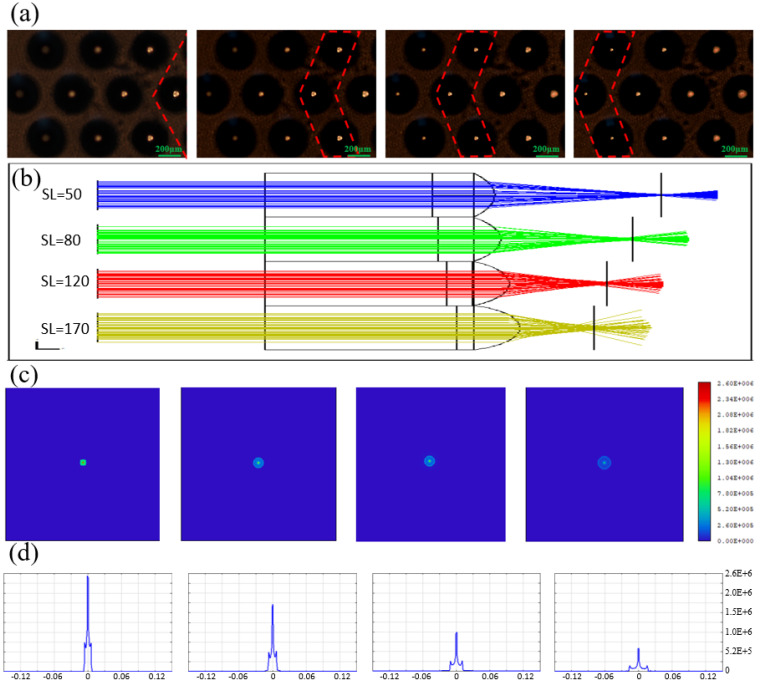
(**a**) Focusing performance of microlens arrays in different focal planes. (**b**) Schematic diagram of simulation experiment. (**c**) Top view of focus energy distribution. (**d**) Cross-sectional view of focal energy distribution.

Similarly, the focusing performance of the above-mentioned submillimeter microlens array was tested. The sample is fixed on a three-dimensional moving platform, and the sample is moved so that the lenses with different NA values are in the best focus position. As shown in Figure 4a, the focal points of each microlens with different NA values are located in different planes, and the smaller the NA value, the larger the focal point diameter.

To further explore the focusing behavior of microlenses with different NA values at the focal point, optical simulation experiments were performed for each lens. As shown in Figure 4b, the four microlenses arranged from top to bottom represent the different processing parameters mentioned above, and their NAs are 0.28, 0.34, 0.39, and 0.44, respectively. The simulation results also show that the microlenses with different NA values have different focal planes. The smaller the NA, the larger the focal length, which is also consistent with the experimental measurement results. To explain the relationship between NA, focal spot size, and brightness at the focal plane of the microlens, we conducted a simulation test of the energy distribution at the focal point, conducting the simulation in the same order as the test order. As shown in Figure 4c, the bright spot at the center of the aperture is the effective imaging area, and its size represents the field of view width of the microlens. In the image, from left to right, the brightness of the central light point gradually decreases, and the area gradually becomes smaller. The larger the size of the bright spot, the larger the imaging area. Theoretically, NA is inversely proportional to the width of the field of view. In the actual measurements, it is manifested by the size of the focal spot. It can be seen that in Figure 4a, the smaller the NA value of the lens, the larger the light spot at the focal point, which is consistent with the simulation results. In addition, for microlenses with different NA values, the smaller the NA value, the higher the energy density at the focal point, which is manifested by the brightness of the focal spot, as shown in Figure 4d. This is the result shown considering the actual loss. This can also be verified in Figure 4a; in the image from left to right, the brightness of the focal point is significantly reduced. In conclusion, the unity of measurement results and simulation results demonstrates the excellent optical performance of the microlenses prepared in this work.

## 4. Conclusions

In conclusion, the microlens array with multiple NAs was fabricated using a femtosecond laser-based linear scanning-assisted wet etching technique. After exploring the influence of laser power, laser scanning speed and scanning length on the microlens, the variation law of the NA of the microlens was obtained. The aperture of the microlens can reach the submillimeter level, and under the same aperture, the NA value can be flexibly adjusted between 0.2 and 0.45. Meanwhile, focusing and imaging performance tests demonstrated the good optical performance of the microlenses, and at the same time demonstrated that it can achieve multi-plane imaging. This processing method gives full play to the flexibility of the femtosecond laser in the field of microlens manufacturing. It provides an effective method of fabricating microlenses with different sizes and NAs, and has application value in the fields of 3D imaging, optical integration, and cell detection.

## Data Availability

Not applicable.

## Supplementary Materials

The following supporting information can be downloaded at: https://www.mdpi.com/article/10.3390/mi13081297/s1, Figure S1: Variation of the cross-sectional profile of a microlens with time; Figure S2: The size of the ablation aperture of the microlenses when they are processed under different laser powers.
