# Supplementary material for: Femtosecond Laser Fabrication of Submillimeter Microlens Arrays with Tunable Numerical Apertures
Source: Micromachines (Basel). 2022 Aug 12;13(8):1297. doi: 10.3390/mi13081297 (PMC9414556; doi:10.3390/mi13081297)
Supplement: Supplementary file 1 [file micromachines-13-01297-s001.zip › micromachines-1803564-supplementary.pdf]

Supplemental Materials

Femtosecond Laser Fabrication of Submillimeter Microlens Arrays with Tunable Numerical Apertures

Tongzhen Yang[1,a], Minjing Li[1,b], Qing Yang[1,*], Yu Lu[2], Yang Cheng[1] , Chengjun Zhang[1], Bing Du[2], Xun Hou[2], and Feng Chen[2,*]

[1] School of Mechanical Engineering, Xi'an Jiaotong University, Xi'an, 710049, PR China
[2] State Key Laboratory for Manufacturing System Engineering and Shaanxi Key Laboratory of Photonics Technology for Information, School of Electronic Science and Engineering, Xi'an Jiaotong University, Xi'an, 710049, PR China
* Correspondence: yangqing@mail.xjtu.edu.cn (Q, Y); chenfeng@mail.xjtu.edu.cn (F, C)
a and b contributed equally to this work;

Supporting Figures.

Figure S1 shows the change process of the cross-sectional profile of the microlens processed with the etching time under the working conditions of a power of 5 mW, a scanning speed of 10 μm/s, and a scanning length of 50 μm. The corrosion times corresponding to the pictures from left to right are 100min, 200min, and 300min in sequence. With the increase of time, its height changes to 55μm, 46μm, 45μm; and its diameter changes to 155μm, 240μm, 310μm.

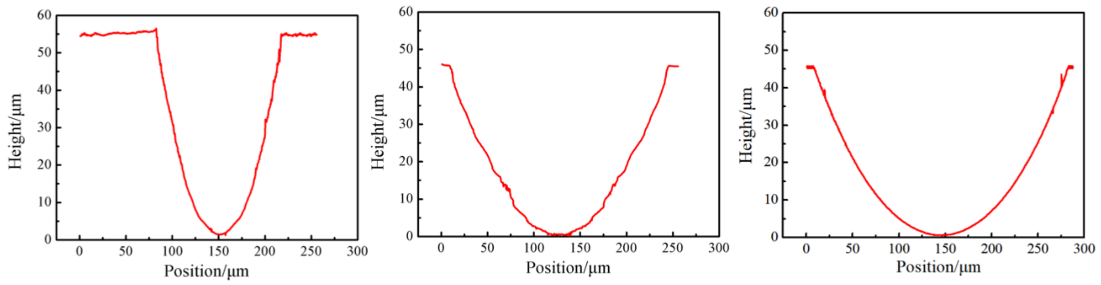

Figure S1. Variation of the cross-sectional profile of a microlens with time.

As can be seen in Figure S2, in the process of increasing power, the diameter of the ablation hole is increasing. But with further increases in power (especially above 6 mW), the asymmetry of the ablated holes also increases. The appearance of this phenomenon will affect the subsequent wet etching process, resulting in the deterioration of the morphology of the sample, and even seriously affect the imaging and focusing performance of the microlens.

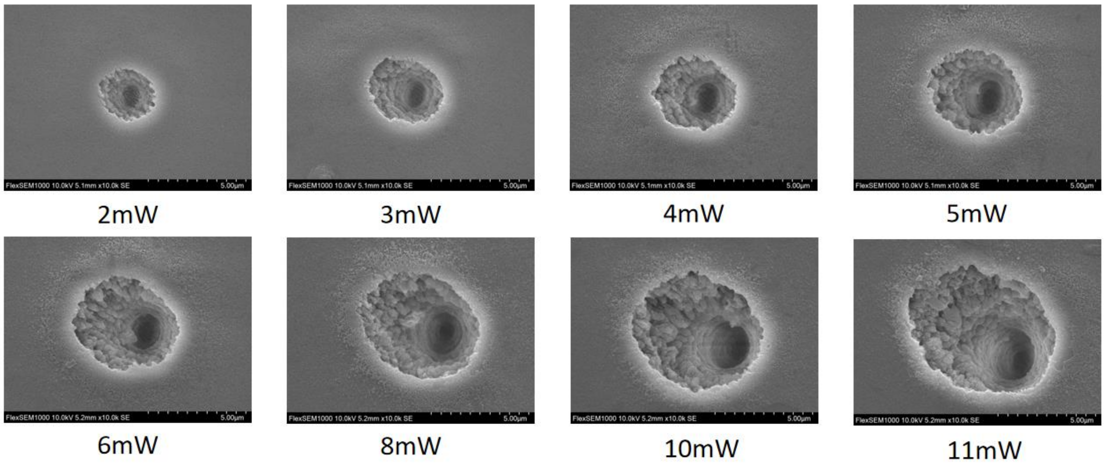

Figure S2. The size of the ablation aperture of the microlenses when they are processed under different laser powers (Taken with scanning electron microscope, same magnification).
